# Preoperative hypocalcemia predicts postoperative complications in older orthopedic patients: A multicenter cohort study

**DOI:** 10.1371/journal.pone.0340876

**Published:** 2026-03-04

**Authors:** Doudou Li, Ting Zhang, Yanmei Ning, Jiacong Miao, Jiana Shi, Ying Hu

**Affiliations:** 1 Clinical Pharmacy Center, Department of Pharmacy, Zhejiang Provincial People’s Hospital, Affiliated People’s Hospital, Hangzhou Medical College, Hangzhou, China; 2 School of Pharmacy, Hangzhou Normal University, Hangzhou, Zhejiang, China; 3 Zhejiang Pharmaceutical University, Ningbo, China; Southern Medical University Nanfang Hospital, CHINA

## Abstract

**Background:**

Serum calcium, a key biochemical marker in the body, plays a crucial role in maintaining bone health. Nevertheless, research exploring the link between preoperative serum calcium levels and the occurrence of postoperative complications in elderly orthopedic patients is currently lacking.

**Aims:**

This study sought to assess the ability of preoperative serum calcium levels to predict the occurrence of postoperative complications in geriatric orthopedic surgery.

**Methods:**

We utilized multivariate logistic regression to identify correlations between serum calcium levels and complications. Generalized additive models to analyze the dose-response relationship with curve fitting and threshold effect evaluation. Subgroup analyses further evaluated the impact of other covariates.

**Results:**

This study included 690 elderly patients undergoing orthopedic surgery. Common postoperative complications primarily included infection, hypoalbuminemia, and electrolyte imbalance, etc. The study demonstrated that preoperative serum calcium levels were an independent protective factor against postoperative complications (OR: 0.24, CI: 0.07–0.76, P = 0.036). When comparing groups based on serum calcium tertiles, patients in the low calcium group exhibited a 79% higher risk of complications compared to the high calcium group (OR = 1.79, 95% CI: 1.12–2.78). Further nonlinear relationship analysis revealed a threshold effect between serum calcium and postoperative complication risk, with a turning point at 2.4 mmol/L. The association was statistically significant below this value but not above it. Subgroup analyses and interaction tests showed that age, gender, comorbidities, and medications, cognitive function, cardiac function, and surgical complexity were not significantly associated with this correlation (P > 0.05 for interaction).

**Conclusion:**

Preoperative calcium screening and correction may represent a simple, low-cost strategy to reduce postoperative complications in elderly orthopedic patients. This study provides evidence for the importance of actively correcting calcium levels before surgery and establishes the value of serum calcium as an early warning indicator for poor prognosis.

## Introduction

With the aging of the population and the increasing prevalence of orthopedic diseases in the elderly population, surgery is often a key tool to relieve pain, rebuild function, and improve quality of life. Hip fracture is the most serious complication of osteoporosis, with about 50 % of patients becoming disabled and 30 % dying within one year of the fracture, making it a common cause of death and disability in older individuals, which in turn generates a heavy family and social burden [1–4]. The incidence of orthopedic diseases and the demand for surgery among older adults are on the rise due to factors such as deterioration of body functions and the combination of various underlying diseases [5–7]. Therefore, how to effectively reduce the occurrence of orthopedic surgical complications has become an urgent problem.

Calcium, as an important electrolyte in the body, regulates a variety of physiological processes, including enzyme activity, gene expression, and cell proliferation [8–10]. It plays a key role in maintaining physiological functions such as muscle contraction, nerve conduction, blood coagulation, and cell signaling [11–13]. However, calcium disorders are prevalent in the clinic, and there is a correlation between blood calcium disorders and complications of severe trauma. Several studies have shown that low blood calcium may increase perioperative blood loss and transfusion rates in elderly hip fracture patients, as since serum calcium, as coagulation factor IV, is a key component of the coagulation cascade reaction [14–16]. In addition, relevant studies have shown that hypocalcemia may affect the central nervous system or cardiovascular system functions and is significantly associated with postoperative morbidity and mortality, with the mechanism is mainly related to the key role of calcium ions in regulating neuronal electrical activity, muscle contraction, and myocardial function [17,18]. Therefore, maintaining stable blood calcium levels is crucial for ensuring homeostatic balance during the perioperative period.

Although serum calcium levels are significantly associated with surgical outcomes, research on their use as prognostic indicators in orthopedic surgery remains very limited. Currently, only one study has focused on preoperative blood calcium levels as a predictor of postoperative mortality in hip fractures [19]. Therefore, this study aims to thoroughly investigate the association between hypocalcemia at admission and the risk of postoperative complications in elderly orthopedic patients, to provide evidence for optimizing perioperative management.

## Materials and methods

### Study design

We conducted a multicenter prospective cohort study from December 20th, 2023, to April 24th, 2025. A total of five centers recruited 690 elderly orthopedic surgery patients. This prospective cohort study was conducted in accordance with the STROBE criteria. The study was approved by the Research Ethics Committee of Zhejiang Provincial People's Hospital (Ethics No. KT2023066) and registered with the China Clinical Trial Registry (Registration No. ChiCTR2300078749). Informed consent was obtained from each patient before data collection.

### Participants

Inclusion criteria:

(1) Orthopedic surgery patients aged ≥60 years;
(2) Patients who chose elective surgery;
(3) Voluntary participation in the study and signing the informed consent form.

Exclusion criteria:

(1) Elderly patients who choose conservative treatment;
(2) Patients who are unable to communicate with others due to illness or mental condition;
(3) Refusing to sign the informed consent form.

### Hospital treatment

Patients were admitted to the hospital and underwent a basic physical examination and blood laboratory tests. Based on the examination results and the patient's physical state, the doctor implemented elective surgical treatment. The main types of surgery include spine surgery, thoracolumbar fusion, arthroplasty, and cervical decompression.

### Quality control

(1) The researchers were strictly trained and familiar with the questionnaire content and communication skills.
(2) Data were entered, checked, and corrected promptly to ensure the reliability of the data.

### Variables

We collected basic information such as age, gender, comorbidity, cardiac function, medication, cognitive function, Surgical complexity, Operation duration, and Intraoperative blood loss based on clinical experience and relevant studies [20–22]. The dependent variable was complication, and the independent variable was preoperative serum calcium level.

### Outcome indicators

The occurrence of postoperative complications is the main outcome indicator. Postoperative complications in this study mainly included swelling, and infection at the surgical site, electrolyte disorders (defined as postoperative blood sodium, potassium, calcium, and chloride above or below the normal laboratory reference values); hypoalbuminemia (serum albumin <30 g/L within 72 hours after surgery); and venous thrombosis (the appearance of bruises and plaques on the lower extremities), which was confirmed by imaging such as ultrasound or CT.

### Statistical analysis

Participants’ serum calcium was classified into three groups: Low (1.82–2.19 mmol/L), Middle (2.19–2.32 mmol/L), and High (2.32–2.75 mmol/L) using the tertile method. Descriptive statistics included mean ± standard deviation for continuous variables, while categorical variables are expressed as percentages. In univariate analyses, group differences were assessed using Student's t-test for normally distributed continuous variables, the Kruskal-Wallis test for non-normally distributed continuous variables, the Chi-square test for categorical variables, and Fisher's exact test when expected frequencies were below 10. We applied the FDR correction to all univariate p-values. Statistical significance was defined as p < 0.05, and corrected p < 0.05 for multiple comparisons.

With serum calcium as the independent variable and the occurrence of complications as the dependent variable, binomial logistic regression was used to analyze the relationship between serum calcium and complications and to understand the confounding factors that may affect it. We constructed three adjusted models using multivariate logistic regression models to analyze the correlation between preoperative serum calcium concentrations and postoperative complications. Serum calcium was transformed into a categorical variable, and sensitivity analyses were performed to verify the stability of the results. Model 1 was not adjusted for covariates. Model 2 is adjusted for age and gender. Model 3 was further adjusted for comorbidity, cognitive function, medication, cardiac function, Surgical complexity, operation duration, and Intraoperative blood loss. We tested for multicollinearity in the fully adjusted model using generalized variance inflation factors. The VIF values for all variables ranged from 1.03 to 1.54, well below the critical criterion of 5, indicating that there is no serious problem of multicollinearity. We performed linear trends by entering blood calcium at each level as a continuous variable in the model. A subgroup analysis of serum calcium and postoperative complications was performed based on criteria such as age, gender, comorbidity, medication, cognitive function, cardiac function, and Surgical complexity. In Model 3, we employed a generalized additive model (GAM) with penalized splines to explore the nonlinear relationship between preoperative serum calcium levels and postoperative complications. The smoothing parameter was optimized automatically by the generalized cross-validation criterion. Subsequently, a two-piece segmented regression model was applied to identify the inflection point. The specific threshold was determined by a grid search algorithm that minimized the residual sum of squares of the model. The statistical significance of this threshold effect was verified by a likelihood ratio test against a simple linear model. Ten of the 690 patients recruited had missing blood calcium levels. These missing values were handled using multiple imputation by random forests (MICE package), under the missing-at-random assumption. This approach was chosen to preserve statistical power and account for the uncertainty associated with imputation. All data analyses were based on R software and Empower Stats software.

## Results

### Patient characteristics

**Fig 1:**
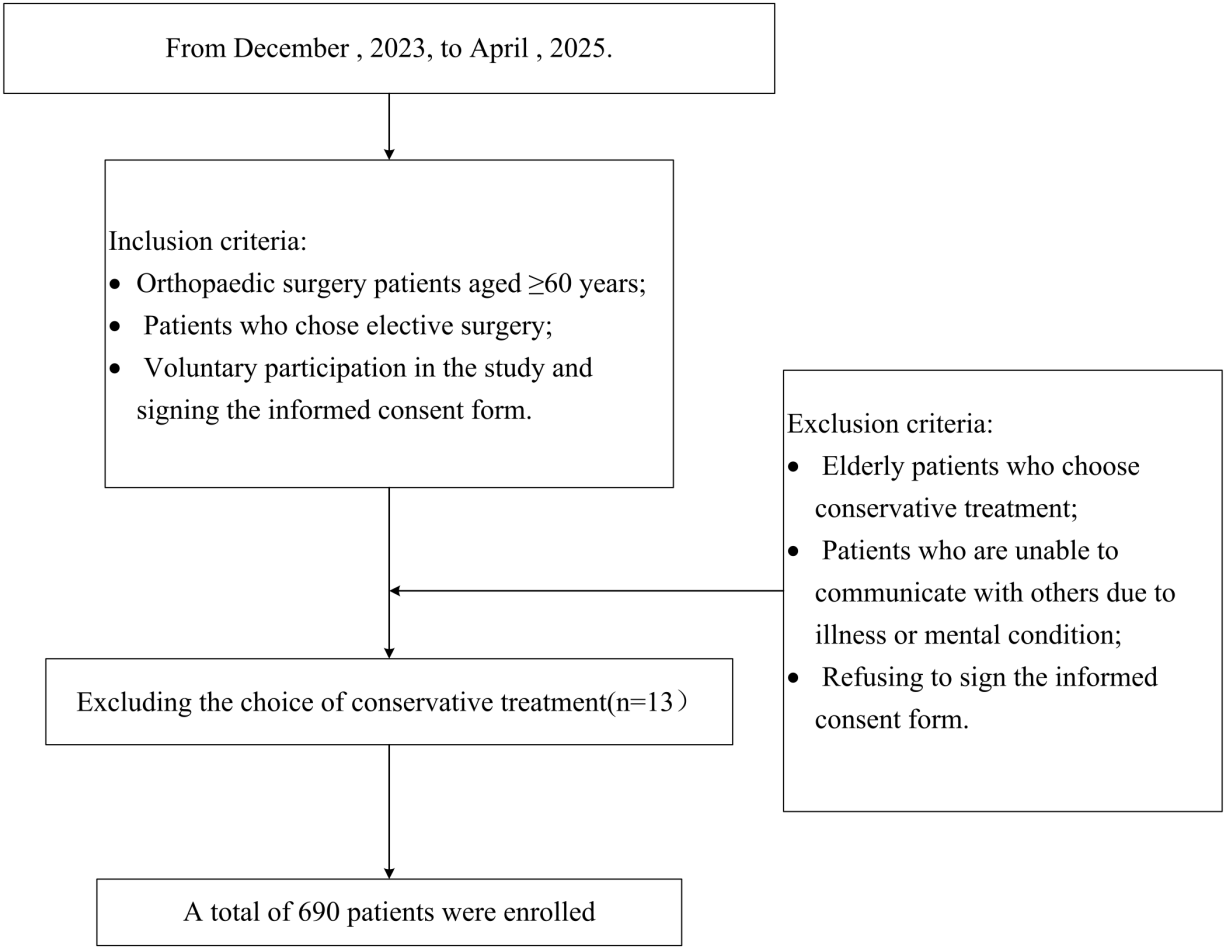
Flow chart of study patients.

A total of 690 elderly patients undergoing orthopedic surgery were enrolled in this study and were followed up for three months postoperatively. The participant selection process is detailed in Fig 1. Serum calcium levels were classified into three groups: low, medium, and high, with 230 in the low group (1.82–2.19 mmol/L), 221 in the medium group (2.19–2.32 mmol/L), and 239 in the high group (2.32–2.75 mmol/L). Postoperative complications occurred in 101 (43.9%) in the low group, 74 (33.5%) in the medium group, and 64 (26.8%) in the high group. Significant differences were observed in serum calcium concerning age, gender, cardiac function, surgical complexity, and complications (P < 0.05). No significant difference was observed in the comorbidities, medications, cognitive function, operation duration, and intraoperative blood loss (p ＞ 0.05). As shown in Table 1.

**Table 1:** Characteristics of the study population.

Characteristic	Calcium tertiles			P-value	P*Value
	Low (1.82–2.19 mmol/L)	Middle (2.19–2.32 mmol/L)	High (2.32–2.75 mmol/L)		
	(n = 230)	(n = 221)	(n = 239)		
**Age(y)**	70.7 ± 7.3	70.7 ± 7.3	68.1 ± 6.3	<0.001	<0.05^*^
**Gender**				<0.001	<0.05^*^
Female	116 (50.4%)	79 (35.7%)	85 (35.6%)		
Male	114 (49.6%)	142 (64.3%)	154 (64.4%)		
**Comorbidity**					
No	74 (32.2%)	83 (37.6%)	72 (30.1%)	0.221	0.316
Yes	156 (67.8%)	138 (62.4%)	167 (69.9%)		
**Cardiac function**				0.003	<0.05^*^
No	172 (74.8%)	185 (83.7%)	207 (86.6%)		
Yes	58 (25.2%)	36 (16.3%)	32 (13.4%)		
**Medication**				0.587	0.587
No	75 (32.6%)	82 (37.1%)	81 (33.9%)		
Yes	155 (67.4%)	139 (62.9%)	158 (66.1%)		
**Cognitive**				0.584	0.587
No	170 (73.9%)	170 (76.9%)	186 (77.8%)		
Yes	60 (26.1%)	51 (23.1%)	53 (22.2%)		
**Operation duration**	1.9 ± 1.3	1.9 ± 1.2	1.8 ± 1.3	0.046	0.076
**Intraoperative blood loss**	61.9 ± 130.3	79.0 ± 233.1	58.4 ± 154.9	0.482	0.587
**Surgical complexity**				0.003	<0.05^*^
Small	67 (29.1%)	74 (33.5%)	110 (46.0%)		
Middle	100 (43.5%)	87 (39.4%)	81 (33.9%)		
Big	63 (27.4%)	60 (27.1%)	48 (20.1%)		
**Complication**				<0.001	<0.05^*^
No	129 (56.1%)	147 (66.5%)	175 (73.2%)		
Yes	101 (43.9%)	74 (33.5%)	64 (26.8%)		

P*: Adjusted P, FDR-corrected p-values;

Adjusted p-values < 0.05 were considered statistically significant.

### Univariate analysis between preoperative serum calcium level and postoperative complications

Binomial logistic regression was used for univariate analysis to determine the relationship between preoperative serum calcium levels and complications and to explore potential confounders. The study showed that age, gender, surgical complexity, operation duration, and intraoperative blood loss were all confounding factors, as shown in Table 2.

**Table 2:** Univariate analysis of serum calcium and postoperative complications.

Risk factors	Statistics, Mean ± SD or n (%)		OR (95% CI)	P Value	P^*^Value
	NO (N = 451)	YES (N = 239)			
**Age**	68.6 ± 6.5	69.9 ± 7.0	1.03(1.00-1.05)	0.016	<0.05^*^
**Gender**					
Female	164 (36.4%)	116 (48.5%)			
Male	287 (63.6%)	123 (51.5%)	1.65(1.20-2.27)	0.003	<0.05^*^
**Serum calcium**	2.3 ± 0.1	2.2 ± 0.1	0.15(0.05-0.47)	0.001	<0.05^*^
**Comorbidity**					
No	154 (34.1%)	75 (31.4%)			
Yes	297 (65.9%)	164 (68.6%)	1.13(0.81-1.59)	0.516	0.554
**Cardiac function**					
No	372 (82.5%)	192 (80.3%)			
Yes	79 (17.5%)	47 (19.7%)	1.15(0.77-1.72)	0.554	0.554
**Medication**					
No	160 (35.5%)	78 (32.6%)			
Yes	291 (64.5%)	161 (67.4%)	1.13(0.81-1.58)	0.508	0.554
**Cognitive**					
No	351 (77.8%)	175 (73.2%)			
Yes	100 (22.2%)	64 (26.8%)	0.78(0.54-1.12)	0.208	0.298
**Surgical complexity**					
Small	218 (48.3%)	33 (13.8%)			
Middle	167 (37%)	101 (42.3%)	4.00(2.57-6.21)	<.001	<0.05^*^
Big	66 (14.6%)	105 (43.9%)	10.51(6.51-16.96)	<.001	
**Operation duration**	1.5 ± 1.0	2.5 ± 1.5	2.03(1.73-2.39)	<.001	<0.05^*^
**Intraoperative blood loss**	35.6 ± 98.0	123.8 ± 259.9	1.01(1.00-1.01)	<.001	<0.05^*^

P*: Adjusted P, FDR-corrected p-values;

Adjusted p-values < 0.05 were considered statistically significant.

### Multivariate analysis between preoperative serum calcium level and postoperative complications

We used three models to explore the relationship between preoperative serum calcium levels and postoperative complications and found significant differences in the three models (P < 0.05). When blood calcium level was treated as a continuous variable, Model 3 showed that for every 1 mmol/L increase in blood calcium level, the risk of complications decreased by 76%. When blood calcium was treated as a categorical variable, compared with the low group, Model 3 indicated that for every 1 mmol/L increase in serum calcium, the risk of complications in the medium group decreased by 34%, which was not statistically significant; while in the high group, the risk of complications decreased by 44%, which was statistically significant. In addition, all three models were tested for trend (P < 0.05), indicating statistical significance, and the results are presented in Table 3.

**Table 3:** Multivariate results by logistic regression.

	Model 1		Model 2		Model 3	
	OR (95%CI)	P, P^*^	OR (95%CI)	P, P^*^	OR (95%CI)	P, P^*^
**Serum calcium**	0.15(0.05,0.47)	P = 0.001 P^*^ < 0.05	0.23(0.07-0.76)	P = 0.016 P^*^ < 0.05	0.24(0.07-0.76)	P = 0.036 P^*^ < 0.05
**Serum calcium tertiles**						
**Low group**	1		1		1	
**Middle group**	0.63(0.43-0.92)	P = 0.016 P^*^ < 0.05	0.72(0.49-1.06)	P = 0.094 P^*^ = 0.094	0.66(0.43-1.01)	P = 0.057 P^*^ = 0.062
						
**High group**	0.46 (0.31-0.69	P = 0.001 P^*^ < 0.05	0.53(0.35-0.79)	P = 0.002 P^*^ < 0.05	0.56(0.36-0.89)	P = 0.014 P^*^ < 0.05
						
**P for trend**	P < 0.001, P^*^ < 0.05		P = 0.002, P^*^ < 0.05		P = 0.012, P^*^ < 0.05	

Model 1: No adjustment.

Model 2: adjusted for age and gender only.

Model 3: Adjusted for age, gender, comorbidity, cardiac function, medication, cognitive function, Surgical complexity, Operation duration, and Intraoperative blood loss.

P^*^: Adjusted P, FDR-corrected p-values;

Adjusted p-values < 0.05 were considered statistically significant.

### Subgroup analysis of serum calcium and postoperative complications

**Fig 2:**
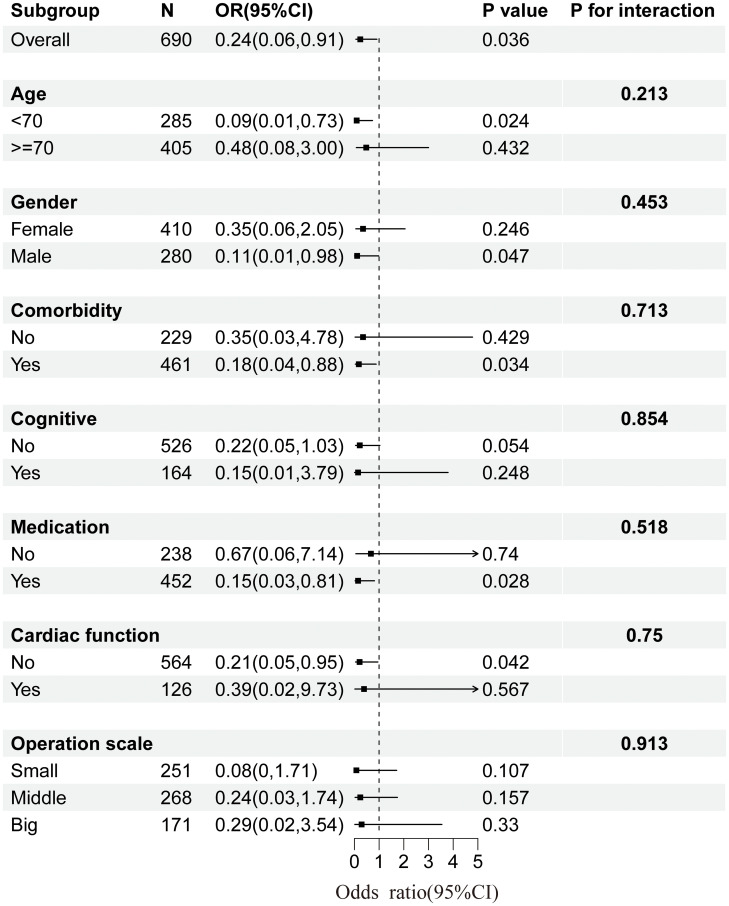
Subgroup analysis plot of serum calcium and complications. Adjusted for age, gender, comorbidity, cardiac function, medication, cognitive function, Surgical complexity, Operation duration, and Intraoperative blood loss. The strata variable was not included in the model when stratifying by itself.

Multiple subgroup analyses and interaction tests based on various covariates were used to assess the robustness of the relationship between serum calcium levels and postoperative complications and to identify potential population differences. The results of the subgroup analyses revealed a consistent relationship between serum calcium and complications in most subgroups, with no significant interaction observed (P > 0.05), as illustrated in Fig 2.

### Curve fitting and threshold analysis

**Fig 3:**
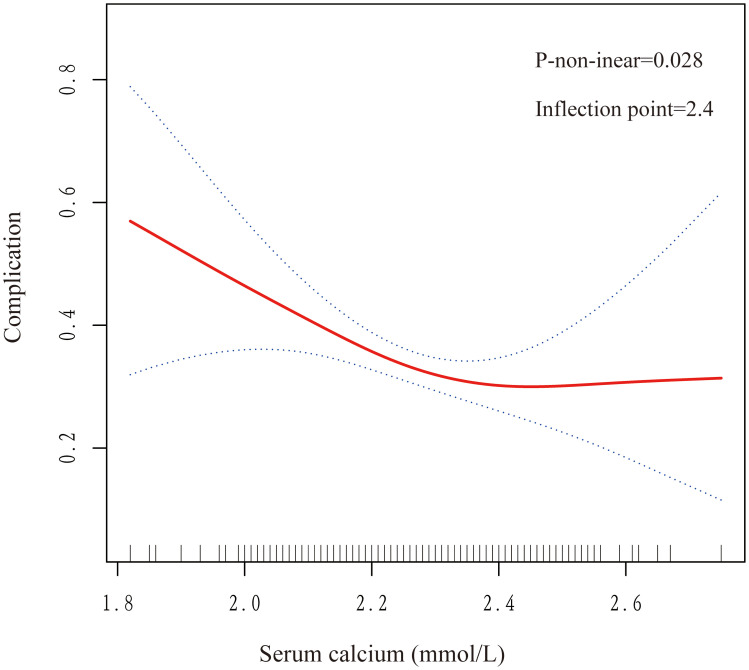
Curve-fit plot of serum calcium levels versus postoperative complications. Values were adjusted for age, gender, comorbidity, cardiac function, medication, cognitive function, Surgical complexity, Operation duration, and Intraoperative blood loss. The solid red line represents the smooth curve fit between variables. Blue bands represent the 95% confidence interval from the fit.

The study performed curve fitting and threshold analysis to further explore the association between serum calcium levels and postoperative complications. The study has revealed a nonlinear relationship between serum calcium levels and postoperative complications, with a p-value of 0.028, as shown in Fig 3. We then used two fitted models to explain this relationship and found an inflection point for the saturation effect. A preoperative serum calcium level of 2.4 mmol/L was the predicted inflection point. When serum calcium was lower than 2.4 mmol/L, the risk of complications was reduced by 90% for every 1 mmol/L increase in blood calcium level (OR 0.1, P = 0.004). When the preoperative calcium concentration was higher than 2.4 mmol/L, the risk of complications tended to stabilize with the increase of blood calcium, but the difference was not statistically significant (OR: 60.0, P = 0.147), as shown in Table 4. Although the normal lower limit of conventional blood calcium is 2.1 mmol/L, for elderly orthopedic patients, 2.4 mmol/L is a more critical level with greater predictive value. Therefore, in clinical management, routine screening and active correction should be carried out for elderly patients with preoperative blood calcium levels below this level to reduce postoperative risks.

**Table 4:** The nonlinearity of preoperative serum calcium vs postoperative complications.

Outcome	OR (95% CI); P Value
**Fitting model by standard linear regression**	0.2 (0.1, 0.9) 0.037
**Fitting model by 2-piecewise linear regression**	
**Inflection point**	
**< 2.4**	0.1 (0.0, 0.5) 0.004
**> 2.4**	60.0 (0.2, 15131.0) 0.147
**P for log-likelihood ratio test**	0.044

The model was adjusted for age, gender, comorbidity, cardiac function, medication, cognitive function, Surgical complexity, operation duration, and Intraoperative blood loss.

Subsequently, smoothing curves were constructed for each age group and gender based on the results of subgroup analysis. Notably, this nonlinear relationship was more pronounced in older patients over 70 years of age and was more prominent in male patients than in female patients (S1 Appendix).

## Discussion

In this multicenter prospective study of older orthopedic patients, we identified a significant nonlinear relationship between preoperative serum calcium levels and the risk of postoperative complications. The inflection point was determined at 2.4 mmol/L. Notably, a preoperative serum calcium level below this threshold was associated with a progressively increasing risk of complications, whereas levels above this point did not confer additional protective benefits. This finding robustly establishes preoperative hypocalcemia as a critical and modifiable risk factor in geriatric orthopedic care. This finding is consistent with a previous study on hip fracture surgery, which observed a similar U-shaped association between preoperative serum calcium levels and postoperative mortality [19]. This study extends the prognostic significance of serum calcium to a wider array of orthopedic procedures in older adults, including fracture repairs, arthritis surgeries, and spinal surgeries. This expanded scope aligns with emerging evidence in other orthopedic subspecialties. A study showed that serum calcium levels are a potential predictor of increased risk of death as a biomarker in the osteoarticular population [23]. Another study showed that in a spinal compression fracture and arthroplasty population, Serum calcium-phosphorus can be used as a serologic indicator to predict the risk of osteoporotic vertebral compression fractures in older adults [24]. The multicenter design employed in this study enhances the generalizability, representativeness, and clinical utility of the findings.

Several interconnected physiological mechanisms may explain the strong association between hypocalcemia and poor surgical outcomes. First, calcium plays a pivotal role in the coagulation cascade and vascular tone. A study in adult trauma patients indicated that low serum calcium levels are correlated with an increased risk of requiring blood transfusions and experiencing anemia [14]. Further research has demonstrated that hypocalcemia can result in greater postoperative blood loss, subsequently elevating the risk of reoperation and mortality [25]. Second, calcium is integral to immune cell function, including neutrophil chemotaxis and phagocytosis. Evidence suggests that hypocalcemia may heighten the risk of surgical site infections, a possible outcome of the impaired regulation of the calcitonin-vitamin D calcium axis due to decreased calcium level [26]. Another study indicates that low calcium levels inherently enhance the risk of electrolyte disturbances. Orthopedic surgeries, due to their substantial fluid requirements, can lead to tissue damage and electrolyte imbalances, significantly affecting postoperative serum electrolyte levels and potentially giving rise to various complications [27]. These factors could represent the underlying mechanisms linking serum calcium levels to orthopedic surgery outcomes, underscoring the critical importance of preoperative serum calcium monitoring in elderly orthopedic patients.

The findings of our study will serve as a reference for the implementation of early calcium supplementation protocols in elderly patients undergoing orthopedic surgery. Given the high prevalence of osteoporosis in this patient demographic, where 64.2% exhibit osteoporotic characteristics and 33.9% experience reduced bone mass, it is evident that dietary calcium intake is inadequate, with only 7.8% of patients meeting or exceeding the daily recommended intake of 1000 milligrams [28].Calcium supplementation is a cornerstone in the management of osteoporosis and is crucial for fracture healing. Evidence indicates that calcium and vitamin D supplementation can significantly enhance fracture healing and improve bone health, leading to a positive impact on the recovery of hip fractures in individuals with osteoporosis [29,30]. Consequently, initiating calcium supplementation promptly may facilitate a more rapid functional recovery for patients. Previous guidelines have focused on the postoperative recovery phase, and patients are usually advised to start monitoring blood calcium levels and taking calcium supplements only after surgery. However, this study found that preoperative calcium supplementation was effective in reducing the risk of postoperative complications in patients with low preoperative calcium levels, and that preoperative monitoring of calcium levels has an important predictive value for surgical prognosis. This is especially true for the patient with a blood calcium of less than 2.4, who should receive early calcium supplementation regardless of the type of orthopedic surgery. Furthermore, our data indicate that this risk may be further exacerbated in males and in elderly patients older than 70 years, a finding not extensively discussed in previous literature. Based on these observations, we propose a stratified treatment strategy for the management of hypocalcemia. For patients with blood calcium levels less than 2.4 mmol/L, conventional treatment is a combination of oral supplemental elemental calcium (1000–1200 mg/day) and vitamin D (800–2000 IU/day) for long-term correction [31]. However, emergency intervention must be initiated when faced with severe hypocalcemia (blood calcium <2.1 mmol/L) or when the condition requires urgent surgery. At this time, intravenous calcium gluconate should be administered under close medical supervision to quickly restore blood calcium levels, usually maintaining supplementation for 24–48 hours. Blood calcium levels should also be closely monitored [32]. Although more data may be needed to further validate this conclusion, our findings have confirmed the importance of preoperative calcium monitoring and regulation. This highlights the need for a balanced approach to calcium supplementation, avoiding both deficiencies and excesses. It provides new ideas and directions for the previous strategy of starting to test blood calcium levels and calcium supplementation only after surgery.

Our study enhances clinical applicability. Involving patients from multiple centers enhances the generalizability and representativeness of our findings. This expansion provides a novel perspective for reducing postoperative complications and optimizing perioperative calcium supplementation strategies in this patient population. Admittedly, this study has several limitations. First, as a prospective multicenter study, our findings lack validation in an external cohort, which necessitates caution when generalizing our results to other populations or healthcare settings. Second, key regulators of calcium homeostasis, such as vitamin D status, renal function, and parathyroid hormone levels, were not measured, and residual confounding from these unmeasured factors may influence the observed associations. Third, our follow-up period was limited to three months, which, while sufficient for capturing most acute postoperative complications, may fail to identify later adverse events. Moreover, the relatively small sample size of patients with blood calcium >2.4 mmol/L limits the statistical power to reliably define risk profiles within this upper range. Future studies should involve larger prospective cohort studies with extended follow-up periods, undergo specialized external validation, and systematically measure calcium and regulatory hormone levels to validate and expand upon our findings.

## Conclusions

Preoperative calcium screening and correction may represent a simple, low-cost strategy to reduce postoperative complications in elderly orthopedic patients. The observed inflection point, with a threshold of 2.4 mmol/L, suggests that this parameter can guide the decision to initiate early calcium supplementation and act as an early warning for potential risks to patient outcomes.

## Supporting information

S1 Appendix Smoothed curve fitting for subgroup analysis. (DOCX)

S2 Appendix TREND statement checklist. (DOCX)

S3 Appendix Study protocol. (DOCX)
