# Supplementary material for: Preoperative hypocalcemia predicts postoperative complications in older orthopedic patients: A multicenter cohort study
Source: PLoS One. 2026 Mar 4;21(3):e0340876. doi: 10.1371/journal.pone.0340876 (PMC12959663; doi:10.1371/journal.pone.0340876)
Supplement: S1 Appendix — (DOCX) [file pone.0340876.s001.docx]

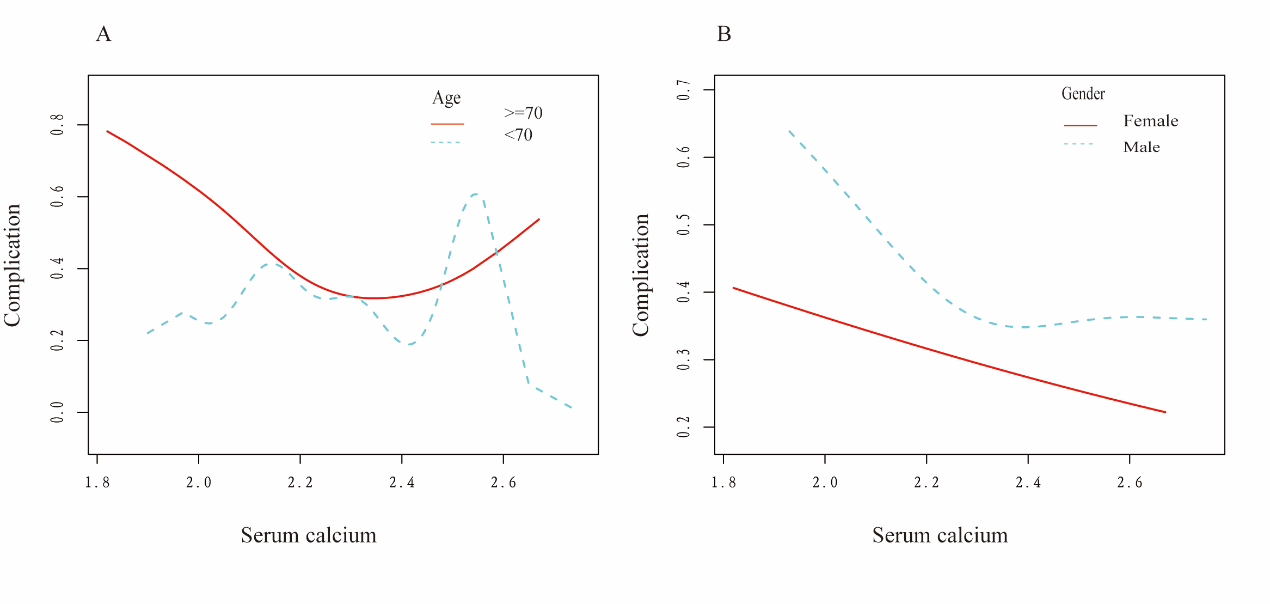


**S1 Appendix.** Smoothed curve fitting for subgroup analysis. A: Curve fitting with age as the subgroup; B: Curve fitting with gender as the subgroup. All adjusted for confounding factors.
