# Supplementary material for: Preoperative hypocalcemia predicts postoperative complications in older orthopedic patients: A multicenter cohort study
Source: PLoS One. 2026 Mar 4;21(3):e0340876. doi: 10.1371/journal.pone.0340876 (PMC12959663; doi:10.1371/journal.pone.0340876)
Supplement: S3 Appendix — (DOCX) [file pone.0340876.s003.docx]

**study protocol**

（Version number: 01 Version date: November 22nd, 2023 ）

**Project Name**: Research on the Construction of a Risk Identification and Prediction Model for Elderly Orthopedic Surgery

**Applicant:** Zhejiang Provincial People's Hospital

**Department responsible for**: Pharmacy Department

**Principal Investigator**: Ying Hu

**Researcher's Declaration and Signature Page of the Plan**

As the main person in charge of this research project, I will abide by the "Ethical Review Measures for Biomedical Research Involving Humans" (2016) issued by the Ministry of Health, the "Helsinki Declaration" (2013) of the World Medical Association, the "International Ethical Guidelines for Human Biomedical Research" (2002) of the Committee on International Medical Ethics, and the ethical principles of Good Clinical Practice. Under the guidance of the quality management norms for drug clinical trials, I will use the approved plan by the ethics committee and conduct the research in accordance with the requirements of this plan to ensure the scientific nature of the research and protect the health and rights of the subjects.

Name：Ying Hu

Date: November 22, 2023

**scenario summary**

| Project Title | Research on the Construction of a Risk Identification and Prediction Model for Geriatric Orthopedic Surgery |
| --- | --- |
| Version number / Version date | 01/November 22nd, 2023 |
| Applicant and Participating Units | Zhejiang Provincial People's Hospital |
| major investigators | Ying Hu |
| Research nature | observational study |
| purpose of research | Through a systematic assessment of the overall condition of elderly patients before orthopedic surgery, a risk identification and prediction model for elderly orthopedic surgeries was established. This model provides a basis for doctors to determine whether elderly frail patients are suitable for elective surgeries and for the comprehensive management of elderly patients during the perioperative period. |
| sample size | 500 |
| object of study | elderly patients undergoing orthopedic surgery |
| research method | incorporate elderly patients undergoing orthopedic surgery, conduct a detailed assessment of their preoperative risk factors, continuously monitor the occurrence of postoperative complications, and follow up on the patients' conditions within 3 months after the surgery. Calculate the incidence of complications and mortality. Based on the assessment of risk factors and the data on complication occurrence and mortality, establish a preoperative risk assessment model for elderly patients undergoing orthopedic surgery. |
| include the criteria | Including 5 hospitals, during the period from December 2023 to December 2024, 500 elderly patients (aged 60 years or older) who were scheduled to undergo orthopedic surgery were selected. The types of surgeries for these patients included hip replacement surgery, knee replacement surgery, spinal compression fractures, fractures of limbs, and other bone and joint surgeries etc. |
| exclusion criteria | 1. Those who do not meet the above-mentioned preoperative diagnostic criteria; ② Patients with multiple fractures accompanied by severe failure of organs such as the brain or heart, kidneys, etc., or who are in critical condition requiring emergency surgery; ③ Patients who have undergone major surgeries within the past 1 month; ④ Patients with severe underlying medical conditions or mental disorders that prevent them from effectively implementing the management plan. |
| Criteria for the end of the experiment | NO |
| Exclusion Criteria | NO |
| Early termination criteria | The participants can voluntarily withdraw or terminate the trial earlier. |
| dosage regimen | NO |
| Primary efficacy indicators | Calculate the incidence rate of postoperative complications in patients |
| Security Index | NO |
| Research Progress Plan | From December 2023 to December 2024, continuous collection and research of data information were carried out.  From January 2025 to April 2025, the data was processed and statistically analyzed to form a model. |
| Statistical analysis methods | Data processing is carried out using the R statistical software. |
| Form of publication of research results | treatise |

**1. Research Objectives**

**1.1 Main Objective**

Through a systematic assessment of the overall condition of elderly patients before orthopedic surgery, a risk identification and prediction model for elderly orthopedic surgeries was established. This model provides a basis for doctors to determine whether elderly frail patients are suitable for elective surgeries and for the comprehensive management of elderly patients during the perioperative period.

**2. Research Background**

Currently, the phenomenon of population aging in our country is intensifying continuously. In 2019, the National Health Commission, the National Development and Reform Commission, and other departments released the "Guiding Opinions on Establishing and Improving the Elderly Health Service System", stating that by 2022, the proportion of secondary and above comprehensive hospitals setting up geriatric medicine departments should reach 50%. They should attach importance to comprehensive assessment of the elderly and the diagnosis and treatment of geriatric syndromes, and promote the transformation of elderly medical services from the single-disease-centered model to the patient-centered multi-disease co-management model. However, at present, in medical institutions in our country, there are relatively few that have established geriatric medicine departments. Most elderly patients still do not receive specialized comprehensive assessments for the elderly and treatment for geriatric syndromes. Especially in terms of surgical treatment, there is a lack of perioperative assessment and management. At present, the risk identification, assessment and management of elderly orthopedic patients are mainly carried out by clinicians based on general preoperative scoring systems in surgery, such as the POSSUM score. The risk level is determined by evaluating the patient's physiological indicators and surgical grade, and then management is carried out. Since such ratings lack specific risk assessment indicators for elderly patients, when patients need to evaluate related issues, they often have to rely on other rating systems, such as the Charlson Comorbidity Index and the Simplified Comorbidity Score. Multiple ratings are complex to operate, and the results are prone to contradictory situations. For elderly patients, conducting a comprehensive assessment of the risks associated with orthopedic surgeries before the operation and taking corresponding solutions are crucial measures to reduce the occurrence of surgical risks and improve the prognosis of patients. Especially, quantifying the surgical risks, formulating scientific and reasonable treatment plans, as well as choosing appropriate treatment methods and timing are of great significance for predicting the prognosis of patients, reducing errors in medical work, and maintaining harmonious doctor-patient relationships. Therefore, this study aims to develop a surgical warning system specifically for elderly patients, with the expectation of providing clinical data references for elderly patients to assess surgical risks and safely navigate the perioperative period.

**3. Test Basis**

**3.1. The preliminary animal experiments and the literature basis of the research**

Take orthopedic surgery as an example. The high incidence of osteoporosis has led to fractures caused by falls among elderly patients, as well as a high prevalence of compression fractures and osteoarthritis. Data from the UK National Statistics show that only 15% of patients undergoing knee and hip joint replacements are under the age of 60, while among hip fractures, the proportion of elderly patients over 60 years old is as high as 90%[1]. Unfortunately, at present, elderly patients do not receive special assessment and management during surgery. The preoperative assessment for them is the same as that for younger patients, and it does not include special indicators for the elderly, such as frailty, cognition, and concurrent medication. There are also no management and rehabilitation treatment measures for special conditions such as frailty in elderly patients after surgery[2-3]. However, at present, elderly patients do not receive special assessment and management during surgery. The preoperative assessment for them is the same as that for younger patients, and it does not include special indicators for the elderly, such as frailty, cognition, and concurrent medication. There are also no management and rehabilitation treatment measures for special conditions such as frailty in elderly patients after surgery[4]. Especially in orthopedic surgeries, taking the surgery for patients with shaft fractures as an example, the probability of death for ordinary patients during treatment is approximately 3.1%. For elderly patients with shaft fractures, the mortality rate within 6 months after injury is 11% to 23%, and the mortality rate within 1 year is 22% to 29%[5]. The persistently high rates of complications and mortality have led to the need for long-term medical treatment for elderly patients after surgery. This not only intensifies the patients' suffering but also causes a continuous increase in medical costs. As a result, clinicians are reluctant to perform surgeries on elderly patients and instead opt for conservative treatments, prolonging the patients' suffering [6]. A study found that only 27% of elderly hip fracture patients in a Beijing tertiary hospital received an assessment by geriatric medicine experts. In contrast, the proportion in the UK National Hip Fracture Database was 70%. Moreover, the mortality rate of elderly hip fracture patients in the UK was significantly lower than that in China [7]. Therefore, it is urgent to develop a comprehensive assessment and management model for the perioperative risks of elderly patients undergoing orthopedic surgeries, to address the problem of high surgical risks and difficult surgical decision-making for elderly patients in our country.

**References：**

1. Bayliss LE, Culliford D, Monk AP, et al. The effect of patient age at intervention on risk pf implant revision after total replacement of the hip or knee: a population-based cohort study[J]. Lancet, 2017,389(10077):1424-1430.
2. Michiel LP， Igande P， Jan-Willem Ｒ， et al. POSSUM predicts hospital mortality and long-term survival in patients with hip fractures[J]． J Trauma， 2011; 42(11): 1043-8.
3. Merad F， Baron G， Pasquet B， et al. Prospective evaluation of in-hospital mortality with the P-POSSUM scoring system in patients undergoing major digestive surgery[J]． World J Surg， 2012; 36(10) : 2320-7．
4. Chong CP， van Gaal WJ， Profitis K， et al． Electrocardiograph changes， troponin levels and cardiac complications after orthopaedic surgery[J]． Ann Acad Med Singap, 2013, 42(1): 24-32．
5. Borges van des Burg BLS, van Dongen TTCF, Moroson JJ, et al. A systematic review and meta-analysis of the use of resuscitative endovascular balloon occlusion of the aorta in the management of major exsanguination[J]. Eur J Trauma Emerg Surg,2018, 44(5):535-550
6. Li Qun, Zhou Chenghong, Liu Jie, et al. Investigation and Analysis of Anxiety Status and Its Influencing Factors in Inpatients with Lower Limb Fractures [J]. Journal of Hunan Normal University (Medical Sciences Edition), 2013, (1): 78-80.
7. Tian M, Gong X, Rath S, Wei J, Yan LL, Lamb SE, Lindley RI, Sherrington C, Willett K, Norton R. Management of hip fractures inolder people in Beijing: a retrospective audit and comparison withevidence-based guidelines and practice in the UK. Osteoporos Int2016;27(2):677–81.

**3.2 Selection criteria for the subjects**

Involving 5 hospitals, during the period from December 2023 to December 2024, 500 elderly patients (aged ≥ 60 years) who were about to undergo orthopedic surgeries were included. The types of surgeries for these patients included hip replacement surgery, knee replacement surgery, spinal compression fractures, fractures of limbs, and other bone and joint surgeries, etc.

Exclusion criteria: ① Those who do not meet the above preoperative diagnostic criteria; ② Patients with multiple fractures accompanied by severe organ failure in the brain or heart, kidneys, etc., or in critical condition requiring emergency surgery; ③ Patients who have undergone major surgeries within the past 1 month; ④ Patients with severe underlying medical conditions or mental disorders that prevent them from effectively implementing the management plan.

**3.3. Dose selection / Administration plan / Dose adjustment basis**

No dose selection / Administration plan / Dose adjustment

**3.4. Basis for endpoint selection**

No

**3.5. Risk and Benefit Basis**

No

**4. Research Content**

**4.1. Trial Population**

**Old patients who were admitted to the hospital for orthopedic surgery**

**4.2. Sample Size Calculation**

A total of approximately 500 orthopedic surgery patients were investigated from December 2023 to December 2024. Since this study involves numerous factors influencing the preoperative assessment, according to the literature opinion in "Sample size calculations: should the emperor's clothes be off the peg or made to measure? (BMJ. 2012 Aug 23 - Zone 1, Impact Factors 105.7), The sample size was calculated using the factor multiplication method. In this study, 25 factors were included, and the sample size was calculated based on a 5-20 times ratio. The commonly used sample size is 250 cases, and the most rigorous sample size is 500 cases.

**4.3 Specific Research Contents**

After the patient was admitted to the hospital, multiple factors such as the patient's basic condition（include heart rate, albumin, electrolyte indicator, serum creatinine, ect.）, past medical history, medication history, self-care ability, comorbidities, cardiac function, cognitive status, and nutritional status were evaluated. At the same time, the surgical size, duration, and intraoperative blood loss during the surgery were also assessed. The occurrence of postoperative in-hospital complications in the patient was tracked and collected. A telephone follow-up was conducted for patient 3 months after the surgery to collect information on the occurrence of postoperative complications and mortality. Analyze the preoperative risk factors and their correlation with postoperative complications and mortality.

**5. Research Methods**

**5.1 Inclusion criteria** (diagnostic criteria, selection criteria, exclusion criteria)

The study included 5 hospitals. During the period from December 2023 to December 2024, 500 elderly patients, aged 60 years or above, who were about to undergo orthopedic surgery, were enrolled. The types of surgeries for the patients included hip replacement surgery, knee replacement surgery, spinal compression fractures, fractures of limbs and other bone and joint surgeries, etc.

Exclusion criteria: ① Those who do not meet the aforementioned preoperative diagnostic criteria; ② Patients with multiple fractures accompanied by severe organ failure (such as in the brain, heart, or kidneys) or critical condition requiring emergency surgery; ③ Patients who have undergone major surgery within the past 1 month; ④ Patients with severe underlying medical conditions or mental disorders that prevent them from effectively following the management plan.

**5.2. Subject grouping**

No grouping

**5.3 Experimental treatment**

Not applicable (No experimental treatment)

**5.4 Criteria for subject early withdrawal/termination of the trial**

The participants can voluntarily withdraw or terminate the trial earlier.

**6. Test Procedure**

**6.1. Subject Management**

1) Method of subject recruitment

All elderly patients who underwent orthopedic surgery in the hospital and met the inclusion/exclusion criteria were included.

2) Informed consent process

Conducted a face-to-face interview with the patients, informed them of the entire research process and possible risks, and invited them to sign the informed consent form.

3) Verification of inclusion/exclusion criteria

Manual verification

4) Examination of medical history and medication records

Manual examination

5) Allocation of screening numbers

No allocation

6) Allocation of treatment/randomization numbers

No grouping

7) Management of trial compliance

No compliance required

**6.2. Safety evaluation procedure** (assessment, detection and reporting of adverse events)

Not applicable

**6.3. Risk Control and Management Procedures**

Not applicable

**6.4.Efficacy measurement procedure**

Not applicable

**6.5. Terminate/Exit the Program**

Not applicable

**6.6. Blinding/Unblinding Procedure**

Not applicable

**6.7. Visitation Requirements**

1) Screening Period

The patient is interviewed in person once to determine eligibility for the study and to collect basic patient information.

2) Treatment Period No

3) Post-treatment visits (safety follow-up visits, follow-up visits, survival follow-up visits)

Telephone follow-ups 3 months after discharge to inquire about the occurrence of complications and readmission situations of the patients.

**7. Start and End of the Trial**

December 2023 - December 2024

**8. Clinical Criteria for Early Termination of the Trial**

No

**9.Data Security and Monitoring Plan**

1) Overview of Data Management Methods

Post-collection batch processing

2) Reporting and collection of adverse events and serious adverse events

Collecting information on all cases

3) Medical Safety Measures

During the perioperative period, routine preoperative and postoperative management is adopted, such as frequent dressing changes and strict aseptic operations. Complications during the perioperative period are handled based on the patient's condition to minimize their occurrence rate.

4) Communication with the Ethics Committee and the superior drug regulatory department

NO

5) Internal data analysis plan

Collect and analyze uniformly

6) Frequency of data security and monitoring reports submitted to the ethics committee

Submit once after completion

**10. Compliance with Ethical Principles and Relevant Regulations**

Excellent

**11. Statistical Analysis Plan**

Data processing will be conducted using R statistical software. Continuous variables are expressed as mean ± standard deviation, while categorical variables are expressed as percentages. For continuous variables, p-values were calculated using the Kruskal-Wallis test. Fisher's exact test was used to determine p-values for categorical variables when the expected count was less than 10.

**12. Publication Form of Research Results**

One research paper will be published.
